# Acanthosis nigricans in a Chinese girl with *FGFR3* K650 T mutation: a case report and literature review

**DOI:** 10.1186/s12881-019-0748-4

**Published:** 2019-01-11

**Authors:** Junling Fu, Yiting Zhao, Tong Wang, Qian Zhang, Xinhua Xiao

**Affiliations:** 1 Department of Endocrinology, Chinese Academy of Medical Sciences and Peking Union Medical College, Peking Union Medical College Hospital, Beijing, China; 2 Department of Center of PET-CT, Chinese Academy of Medical Sciences Cancer Institute and Hospital, Beijing, 100021 China

**Keywords:** Acanthosis nigricans, *FGFR3*, Mutation

## Abstract

**Background:**

Acanthosis nigricans (AN) is a clinical manifestation featured by velvety brown plaques in skin folds that occurs in some hereditary and syndromic disorders. Fibroblast growth factor receptor 3 (*FGFR3*) mutations have been identified as one of the genetic causes of inherited AN.

**Case presentation:**

A 17-year-old Chinese female had presented generalized acanthosis nigricans since she was 4 years old. She yielded no family history of short stature or AN. Apart from a short stature, no skeletal defects, neurological defects or other abnormalities were found. To identify the aetiology of the clinically diagnosed AN, we screened the proband for genetic mutations using whole exome sequencing. A heterozygous mutation (c.1949A > C, p.Lys650Thr) in *FGFR3* was found in the proband. To date, 26 cases of AN harbouring this specific gene mutation have been reported in the literature, and only one child carried a de novo mutation instead of inheriting the specific mutation from their parents. The present case is the first-reported Chinese patient with isolated AN with a de novo K650 T mutation in *FGFR3*.

**Conclusions:**

We reported a new case of AN caused by a heterozygous mutation (c.1949A > C, p.K650 T) in *FGFR3*, and review the past reports of AN with the same gene mutation. Sequencing of the *FGFR3* gene is a feasible approach to identify the aetiology of AN, especially for early onset extensive AN.

**Electronic supplementary material:**

The online version of this article (10.1186/s12881-019-0748-4) contains supplementary material, which is available to authorized users.

**Electronic supplementary material:**

The online version of this article (10.1186/s12881-019-0748-4) contains supplementary material, which is available to authorized users.

## Background

Acanthosis nigricans (AN) is characterized by velvety and pigmented hyperkeratosis of the skin, primarily in the skin folds of the neck, armpits, and groin. AN can occur as a symptomatic state of several genetic diseases, many of which are caused by insulin resistance syndromes or functional aberration of FGFR [1]. Mutations in fibroblast growth factor receptor 3 (*FGFR3*) are known to cause several kinds of skeletal dysplasia accompanying AN [2–5].

*FGFR3* encodes a member of the fibroblast growth factor receptor (FGFR) family which is comprised of four related receptors (FGFR1–4) [2]. Different mutations in *FGFR3* have been identified in patients with hypochondroplasia (HCH) [2–5]. Additionally, AN has occasionally been reported in patients with mutations in *FGFR3* [1–6]. In 2007, the first familial case of AN was reported with heterozygous mutation at codon 650 (p. Lys650Thr) in *FGFR3* [6]. Presently, there have been 6 reports, totaling 26 cases, that have shown this specific gene mutation in AN, and only one child carried a de novo mutation instead of inheriting the specific mutation from their parents [1–6]. In this study, we describe the first Chinese clinically diagnosed AN case with a de novo *FGFR3* mutation (p. Lys650Thr), and review the previous reports of AN associated with activating mutations of *FGFR3*.

## Case presentation

A 17-year-old girl was referred to our endocrinology clinic for hyperkeratotic and pigmented lesions on her neck and whole trunk, which initially appeared when she was 4 years old. Her height was within the normal range during childhood (< 4 years) but gradually began to be under the normal growth curve, ultimately resulting in grown-up short stature.

**Fig. 1:**
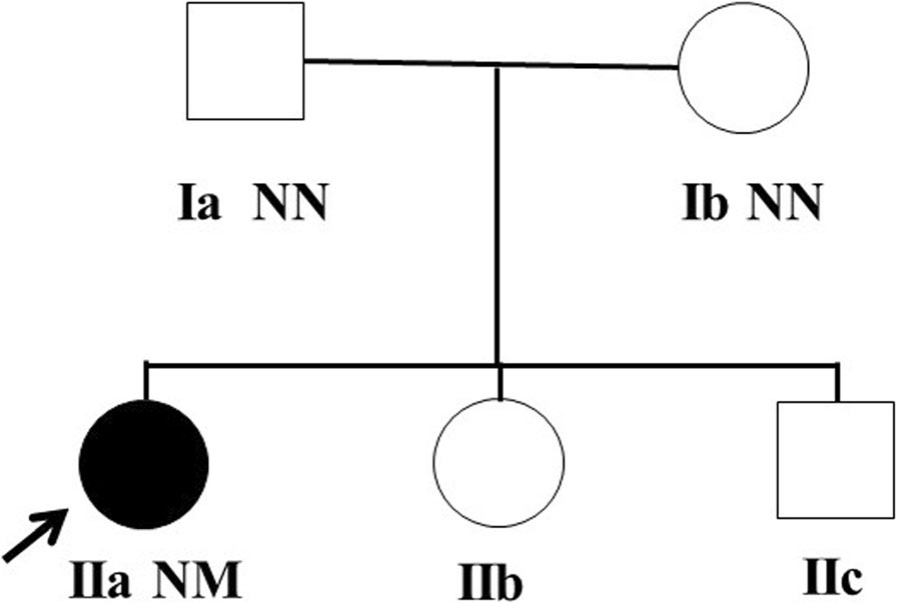
Pedigree of family. Squares represent male family members, while circles represent female family members. Black symbol represents individual with acanthosis nigricans, blank symbols represent normal individuals. Arrow indicates proband in the family (IIa). Variant carrier status present as N: Normal allele and M: Mutation. The sequence data displayed heterozygous mutation in *FGFR3* (c.1949A > C, p.Lys650Thr) in the proband (IIa)

The patient was the first child of an unrelated Chinese couple. Her mother underwent vaginal delivery after a full-term pregnancy. The birth weight of the girl was 4 kg and the birth length was 50 cm. She exhibited no neurological defects or skeletal abnormalities, no diabetes mellitus or its related symptoms, and no family history of cancer. The patient’s parents, younger sister and brother had no significant medical history (Fig. 1).

**Fig. 2:**
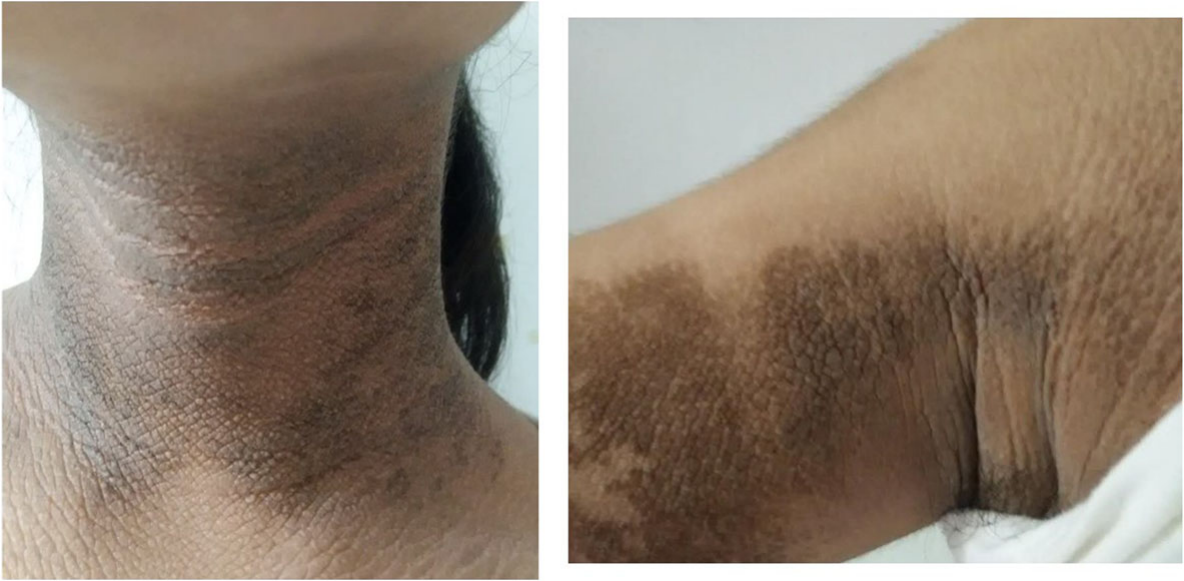
Clinical images of the neck, and axillary fossa region of the case

On physical examination, the patient exhibited extensive, velvety, thick, hyperpigmented plaques involving the neck, back, and axillae (Fig. 2). The patient was a non-dysmorphic girl with the height of 146 cm (<-2SD).

Laboratory tests revealed no abnormal biochemical findings (Table 1). The thyroid hormone, cortisol and androgen levels were within the normal range (the testosterone level demonstrated in Table 1 was under the reference range, we tested testosterone one more time, and the other value was normal: 31.8 ng/dl). Fasting blood glucose and fasting insulin level were 88.2 mg/dL and 13.78μU/ml, respectively. The homeostasis assessment index for insulin resistance (HOMA-IR) as the outcome of the fasting insulin (mUI/ml) × glucose (mmol/l) /22.5 was 3.0. This result indicated no insulin resistance. These findings excluded the diagnosis of insulin resistance, T2D, Cushing’s syndrome and hyperandrogenism.

**Table 1:** Laboratory investigation

Laboratory (serum)	Value	Normal range
Alanine transaminase (U/L)	8	7–40
Aspartate aminotransferase (U/L)	16	13–35
Total bilirubin (umol/L)	9.1	5.1–22.2
Direct bilirubin (umol/L)	3.5	0–6.8
Albumin (g/L)	48	35–52
Creatinine (umol/L)	57	18–69
Uric acid (umol/L)	337	150–357
Total cholesterol (mmol/L)	4.03	2.85–5.7
Triglyceride (mmol/L)	0.36	0.45–1.70
HDL-C (mmol/L)	1.38	0.93–1.81
LDL-C (mmol/L)	2.24	< 3.37
Fating glucose (mmol/L)	4.9	3.9–6.1
Fasting insulin (uIU/ml)	13.78	5.2–17.2
HOMA-IR	3.0	< 3.0
Glycosylatedhemoglobin (%)	5.1	4.5–6.3
Homocysteine (umol/L)	10.3	< 15
hsCRP (mg/L)	0.12	0–3.0
Erythrocyte sedimentation rate (mm/h)	5	0–20
Cortisol (ug/dl)	22.06	4–22.3
Testosterone (ng/dl)	17.3	25.6–42.6
DHES (ug/dl)	161.3	17–343
17aOHP (ng/ml)	1.04	0.27–2.9
FT3 (pg/ml)	3.27	1.8–4.1
FT4 (ng/dl)	1.231	0.81–1.89
TSH (uIU/ml)	3.242	0.38–4.34
A-Tg (IU/ml)	< 10	< 115
A-TPO (IU/ml)	< 5	< 34

Abbreviation: *HDL-C* high-density lipoprotein cholesterol, *LDL-C* low-density lipoprotein cholesterol, *HOMA-IR* homeostasis model assessment of insulin resistance, *hsCRP* high sensitivity C reactive protein, *DHES* dehydroepiandrosterone sulfate

**Fig. 3:**
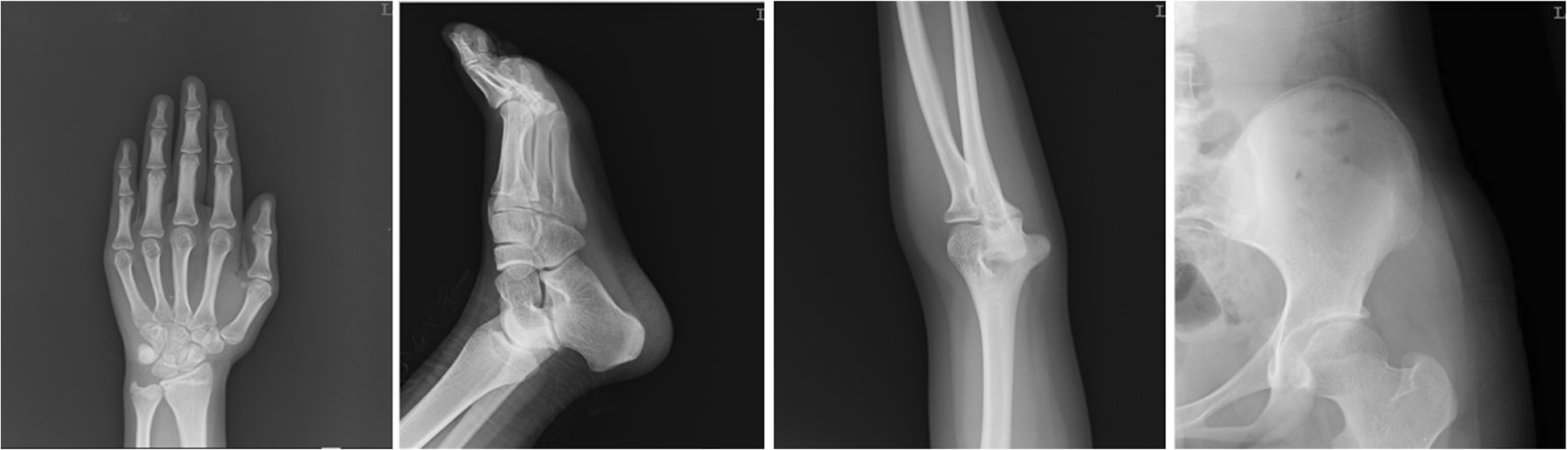
X ray of the case (done at 14 years old, left side)

X-ray examination (done at 14 years old) revealed no abnormalities (Fig. 3).

As genetic mutations have been recognized in several cases of syndromic AN, a mutational analysis was performed in the proband and parents. Written informed consent was signed by the proband and her parents.

### Genetic analysis

Peripheral blood samples (4 ml) of the proband and her parents were collected. Genomic DNA was extracted from the blood using a QIAamp DNA Mini Kit (Qiagen China Co., Ltd., Shanghai, China) according to the manufacturer’s recommendations. We first performed whole exome sequencing for the proband. Next, based on the test results of whole exome sequencing, the presence of the mutation in the proband and her parents was confirmed with direct Sanger sequencing of the affected exon.

### Whole exome sequencing

All coding exons were enriched using the xGen Exome Research Panel v1.0 (Integrated DNA Technology, Inc). Captured DNA libraries were sequenced on Illumina Hiseq X Ten according to the manufacturer’s instructions for paired-end 150 bp reads. Variants were considered as pathogenic mutations if they exhibited the following components: i) rare or absent in the above genome databases; ii) variation expected to have a drastic effect on the protein (nonsense mutation, frame shift mutation, mutation at a splice site, or missense mutation is highly conserved among species); and iii) variation predicted to be damaging.

### Sanger sequencing to validate

Sanger sequencing of the affected exon in *FGFR3* was performed on DNA samples from the proband and her parents. According to the DNA sequence of the *FGFR3* gene, primers of exon 14 of *FGFR3* were designed using Primer Premier 5 software. The functional effects of protein variants were predicted by PolyPhen2 (http://genetics.bwh.harvard.edu/pph2/), SIFT (http://sift.jcvi.org) and Mutation Taster (http://www.mutationtaster.org).

**Fig. 4:**
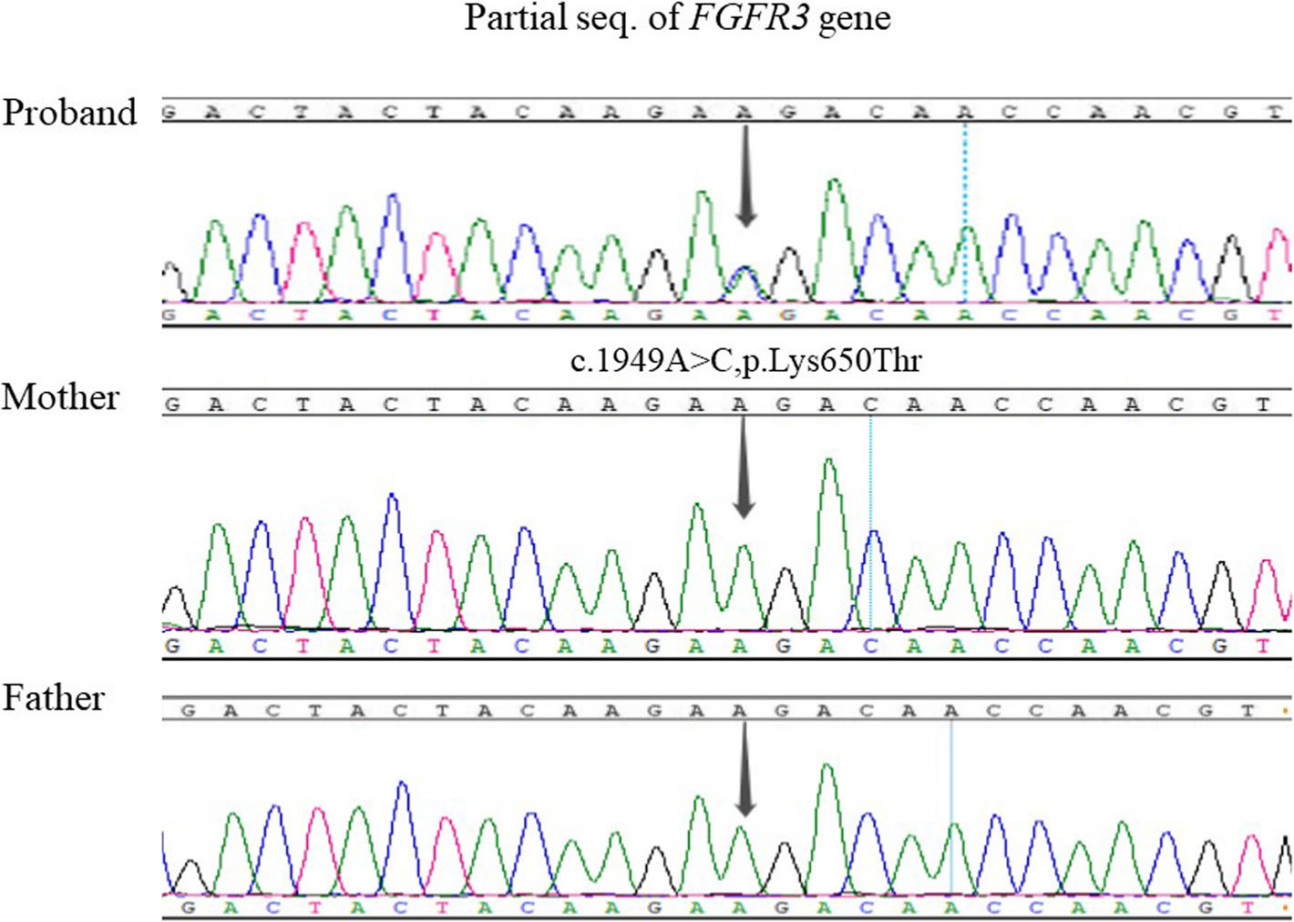
Sequencing of exon 14 of *FGFR3* (NM_000142). A heterozygous c.1949A > C (p.Lys650Thr) at codon 650 of *FGFR3*gene was revealed in the proband, while not in her unaffected parents

Through data mining, combined with genetic characteristics and clinical manifestations, we identified a heterozygous c.1949A > C, p.Lys650Thr mutation in *FGFR3* of the proband, which is considered to be a pathogenic mutation. As the proband’s parents did not carry the mutation, the mutation identified in the proband was a de novo mutation. Sanger sequencing confirmation is shown in Fig. 4. The mutation caused change in the protein from K to T at p. Lys650, which is located in exon 14 of *FGFR3.* The pathogenicity of the mutation on bone and skin has been previously reported [1–6] and was confirmed using 3 different software programmes (The expected score scales of the mutation from each software programme are shown in Additional file 1: Table S1): SIFT (0), PolyPhen-2 (1) and Mutation taster (disease-causing).

## Discussion and conclusions

In this study, we have identified the first Chinese general AN case caused by the mutation in *FGFR3* (c.1949A > C, p.Lys650Thr). Additionally, we reviewed the previously reported cases due to this mutation.

AN is characterized by dark-brownish hyperpigmentation, velvety with thickening of the skin, and the skin lesions are usually founded in skin folds such as the neck, armpits, forehead, and groin. The exact incidence of AN is still unknown. AN’s prevalence in whites is less than 1%, whereas the prevalence is higher in dark-skinned people, approximately 13.3% [7]. AN in childhood is not rare and the benign hereditary form and the type associated with insulin resistance are commonly seen [8]. However, in the present case, the patient did not have insulin resistance or a family history of AN. Additionally, AN is correlated with numerous genetic syndrome, which can be divided into insulin resistance syndromes and fibroblast growth factor (FGF) defects [9]. The pathogenic variant (p.Lys650Thr) in *FGFR3* has been recognized to be correlated with AN [1–6]. The present case is the first-reported Chinese patient having AN with this specific mutation.

*FGFR3* is located at 4p16.3, and encodes the fibroblast growth factor receptor 3 [6]. The gene is highly conserved between members, and acts as a repressor of long bones growth [6]. The full-length FGFR3 protein consists of an extracellular region, a single hydrophobic membrane-spanning segment and a cytoplasmic tyrosine kinase domain. The extracellular portion of the protein combined with fibroblast growth factors; set in motion a cascade of downstream signals, influencing mitogenesis and differentiation, and ultimately playing a role in the development and maintenance of bone. Mutations in this gene lead to craniosynostosis and multiple types of skeletal dysplasia (https://www.ncbi.nlm.nih.gov/protein/4503711). Located in the tyrosine kinase domain II of *FGFR3*, P. Lys650 is an important residue for the biological function of *FGFR3* [2]. Germline K650 T mutation can results in constitutive activation of *FGFR3* signals through the activation of STAT1 and MEK/MAPK pathways, both of which are described relevant to the phenotypic consequences of skeletal dysplasia [10]. However, the exact mechanisms of the association between AN and the K650 T mutation have not been thoroughly elucidated to date. Given that HCH accompanying AN happens in subjects with p.Lys650Thr mutation, it has been speculated that the activation of the MAPK pathway (affecting proliferation of keratinocytes) and PI3-K/Akt pathway (affecting the expansion of the epidermal compartment) may play a role in the development of AN [2].

We reviewed the previously reported cases caused by a mutation in *FGFR3.* The specific mutation in *FGFR3* was first reported in a family that included 4 patients diagnosed with AN without apparent skeletal deformity [6]. Currently, 7 pedigrees containing 27 cases (including the current case) have been described (Table 2) [1–6]. The reviewed cases yield no gender differences, and the male to female ratio was 13:14. The height of all of the patients tends to be low, which is consistent with the case presented in our study. The majority of the individuals showed excessive skin pigmentation without obesity or diabetes mellitus (with the exception of patient 5 who was diagnosed with adult onset diabetes mellitus) during infancy. Moreover, 19 of the 27 cases were diagnosed as HCH plus AN, while several cases were described as “pure” familial AN (*n* = 8). Additionally, apart from the familial AN, few cases harbor a de novo mutation [4]. In our study, neither of the parents of the patient carried the *FGFR3* mutation, indicating a de novo mutation in the proband.

**Table 2:** Reports on AN with or without HCH due to p. Lys650Thr of *FGFR3*

Patients	Onset age	Age	Gender	AN	HCH	Birth weight (g)	Height (cm)	BMI	FBG(74-105 mg/dL)	FINS (1.5–18.5μU/ml)	HOMA-IR	HbA1c
Family 3[6]												
1^a^	infancy	4	F	+	–	/	105.5 (5.5y,8th)	15.5	normal	/	/	normal
2	/	25	M	+	–	/	156	19.7	/	/	/	/
3	/	29	F	+	–	/	145 (<5th)	30.5	/	/	/	/
4	/	11	F	+	–	/	139 (26th)	21.2	/	/	/	/
Family 1[2]												
5	infancy	51	M	+	+	/	144.3	26.4	190	5.3	2.4	7.9
6	infancy	49	F	+	+	/	141.2	28.1	81	3.6	0.7	4.4
7	infancy	46	F	+	+	/	145.5	26.9	92	4.4	0.9	4.9
8	infancy	40	F	+	+	/	149.4	28.3	87	5.1	1.1	4.8
9	infancy	35	F	+	+	/	153	25.8	85	3.9	0.8	4.3
10^a^	infancy	16	M	+	+	3515	/	24.2	97	7.0	1.6	4.6
11	infancy	18	F	+	+	/	147.9	26.5	73	2.8	0.5	4.2
12	infancy	8	M	+	+	/	/	20.6	88	3.8	0.8	4.5
13	infancy	13	M	+	+	/	/	20.2	90	6.9	1.5	4.6
14	infancy	7	M	+	+	/	/	23	92	6.1	1.3	4.7
Family 5[1]												
15^a^	3	15	F	+	–	/	/	/	/	/	/	/
16	/	/	F	+	–	/	/	/	/	/	/	/
17	/	/	F	+	–	/	/	/	/	/	/	/
Family 2[3]												
18^a^	12	14	M	+	+	/	143	21.5	/	/	/	/
19	/	/	M	+	+	/	/	/	/	/	/	/
20	/	/	M	+	+	/	/	/	/	/	/	/
21	/	/	M	+	+	/	/	/	/	/	/	/
Family 4[4]												
22^a^	1	3	M	+	+	2883	91.7 (3-10th)	19.4	76	8.8	1.6	
Family 6[5]												
23^a^	2	10	M	+	+	/	128	23.1	/	3.1	/	/
24	/	12	F	+	+	/	135	23.5	/	/	/	/
25	/	47	M	+	+	/	155	25	/	/	/	/
26	/	74	F	+	+	/	140	23	/	/	/	/
Current case												
27^a^	4	17	F	+	–	4000	146	24.4	88.2	13.78	3.0	5.1

^a^Proband of the family

*AN* acanthosis nigricans, *HCH* hypochondroplasia, *BMI* body mass index, *FBG* fasting glucose, *FINS* fasting insulin, *HOMA-IR* homeostasis model assessment index of insulin resistance, *HbA1c* glycosylated hemoglobin

The treatment of AN should focus on correction of the underlying pathological state. Currently, there is no good treatment for AN caused by a mutation in *FGFR3*. It may be necessary for the present patient to monitor the levels of fasting blood glucose and androgen regularly.

In summary, we report the first Chinese case of AN with p.Lys650Thr mutation in *FGFR3*, demonstrating a widespread skin pigmentation, and short stature. *FGFR3* sequencing is a feasible approach to identify the aetiology of AN, and the effects of *FGFR3* on bone and skin should be further analysed.

## Additional file

Additional file 1: **Table S1.** The expected score scale of the mutation from each software. (DOCX 13 kb)

## Abbreviations

AN: Acanthosis nigricans
*FGFR3*: Fibroblast growth factor receptor 3
HCH: Hypochondroplasia
