# Supplementary material for: Acanthosis nigricans in a Chinese girl with FGFR3 K650 T mutation: a case report and literature review
Source: BMC Med Genet. 2019 Jan 11;20:8. doi: 10.1186/s12881-019-0748-4 (PMC6329052; doi:10.1186/s12881-019-0748-4)
Supplement: Supplementary file 1 — Table S1. The expected score scale of the mutation from each software. (DOCX 13 kb) [file 12881_2019_748_MOESM1_ESM.docx]

| **Supplementary Table 1. The expected score scale of the mutation from each software** | | | |
| --- | --- | --- | --- |
| Software | Score | Predictive pathogenicity | Threshold/interpretation |
| SIFT | 0 | D | D: Deleterious (sift ≤ 0.05) |
|  |  |  | T: Tolerated (sift＞0.05) |
| Polyphen2_HDIV | 1.0 | D | D: Probably damaging (≥ 0.957） |
|  |  |  | P: Possibly damaging (0.453-0.956) |
|  |  |  | B: Benign (≤ 0.452) |
| Polyphen2_HVAR | 1.0 | D | D: Probably damaging (≥ 0.957) |
|  |  |  | P: Possibly damaging (0.453-0.956) |
|  |  |  | B: Benign (≤ 0.452） |
